# Antiretroviral exposure and comorbidities in an aging HIV-infected population: The challenge of geriatric patients

**DOI:** 10.1371/journal.pone.0203895

**Published:** 2018-09-21

**Authors:** Clotilde Allavena, Matthieu Hanf, David Rey, Claudine Duvivier, Firouze BaniSadr, Isabelle Poizot-Martin, Christine Jacomet, Pascal Pugliese, Pierre Delobel, Christine Katlama, Véronique Joly, Christian Chidiac, Nathalie Dournon, Dominique Merrien, Thierry May, Jacques Reynes, Amandine Gagneux-Brunon, Catherine Chirouze, Thomas Huleux, André Cabié, François Raffi, the Dat’AIDS study group

**Affiliations:** 1 Infectious Diseases Department, University Hospital of Nantes, Nantes, France; 2 INSERM CIC1413, University Hospital of Nantes, Nantes, France; 3 INSERM UMR 1181 B2PHI, Versailles Saint Quentin University, institut Pasteur, Villejuif, France; 4 Centre for HIV Infection Care, Strasbourg, France; 5 AP-HP-Necker Hospital, Infectious Diseases Department, Necker-Pasteur Infectiology Centre, Paris, France; 6 Medical Centre of Pasteur Institut, Necker-Pasteur Infectiology Centre, Paris, France; 7 EA7327, Paris Descartes University, Sorbonne Paris Cité, Paris, France; 8 Department of Internal Medicine, Infectious Diseases, and Clinical Immunology, Reims Teaching Hospitals, University of Reims, Reims, France; 9 Université de Reims Champagne-Ardenne, Faculté de médecine, EA-4684 / SFR CAP-SANTE, Reims, France; 10 Immuno-Hematology Clinic, Aix-Marseille University, APHM Hôpital Sainte-Marguerite, Marseille, France; 11 Inserm U912 (SESSTIM), Marseille, France; 12 Infectious Diseases Department, University of Clermont-Ferrand, Clermont-Ferrand, France; 13 Department of Infectious Diseases, Centre Hospitalier Universitaire de l'Archet, Nice, France; 14 INSERM, UMR1043, Toulouse and Université Toulouse III Paul Sabatier, Toulouse, France; 15 Department of Infectious Diseases, Toulouse University Hospital, Toulouse, France; 16 Department of Infectious Diseases, Assistance publique-Hôpitaux de Paris, Pitié-Salpêtrière University Hospital, Paris, France; 17 Inserm Unité Mixte de Recherche en Santé 1136, Université Pierre et Marie Curie Paris 06, Sorbonne Universités, Paris, France; 18 Infectious Diseases Department, Hôpital Bichat, AP-HP, Paris, France; 19 National Institute of Health and Medical Research (INSERM) IAME, UMR 1137, Paris Diderot University, Sorbonne Paris Cité, Paris, France; 20 Infectious and Tropical Diseases Department, Hospices Civils de Lyon, Claude Bernard Lyon 1 University, Lyon, France; 21 Service de Maladies Infectieuses et Tropicales, Dermatologie, Médecine Interne, Faculté de Médecine Hyacinthe Bastaraud, Université des Antilles, Pointe à Pitre, France; 22 Inserm CIC 1424, Centre Hospitalier Universitaire de Pointe-à-Pitre, Pointe-à-Pitre, France; 23 Departement of infectious diseases, CHD Vendee, La Roche sur yon, France; 24 Department of infectious diseases, University Hospital Centre, Nancy, France; 25 Infectious Diseases Department, Montpellier University Hospital, Montpellier, France; 26 UMI233 INSERM U1175, Montpellier University Hospital, Montpellier, France; 27 Infectious Diseases Department, University of Saint-Etienne, Saint-Etienne, France; 28 Infectious Diseases Department, University hospital of Besançon, Besançon, France; 29 UMR CNRS 6249, University of Bourgogne-Franche Comté, Besançon, France; 30 Infectious Diseases Department, University hospital of Tourcoing, Tourcoing, France; 31 Infectious Diseases Department, University Hospital of Martinique, Fort-de-France, France; 32 EA4537, Université des Antilles, Fort-de-France, France; Katholieke Universiteit Leuven Rega Institute for Medical Research, BELGIUM

## Abstract

As HIV-infected adults on successful antiretroviral therapy (ART) are expected to have close to normal lifespans, they will increasingly develop age-related comorbidities. The objective of this cross-sectional study was to compare in the French Dat’AIDS cohort, the HIV geriatric population, aged 75 years and over, to the elderly one, aged from 50 to 74 years. As of Dec 2015, 16,436 subjects (43.8% of the French Dat’AIDS cohort) were aged from 50 to 74 (elderly group) and 572 subjects (1.5%) were aged 75 and over (geriatric group). Durations of HIV infection and of ART were slightly but significantly different, median at 19 and 18 years, and 15 and 16 years in the elderly and geriatric group, respectively. The geriatric group was more frequently at CDC stage C and had a lower nadir CD4. This group had been more exposed to first generation protease inhibitors and thymidine analogues. Despite similar virologic suppression, type of ART at the last visit significantly differed between the 2 groups: triple ART in 74% versus 68.2%, ART ≥ 4 drugs in 4.7% versus 2.7%; dual therapy in 11.6% versus 16.4% in the elderly group and the geriatric group, respectively. In the geriatric group all co-morbidities were significantly more frequent, except dyslipidemia, 4.3% of the elderly group had ≥4 co-morbidities versus18.4% in the geriatric group. Despite more co-morbidities and more advanced HIV infection the geriatric population achieve similar high rate of virologic suppression than the elderly population. A multidisciplinary approach should be developed to face the incoming challenge of aging HIV population.

## Introduction

As a benefit of long term suppressive antiretroviral therapy with improved tolerability, the number of aging HIV infected individuals is increasing with about half of them above 50 years of age or over in high-income countries [1, 2]. Both life expectancy and mortality rates improved over time linked with coverage improvement, quality and tolerability of ART regimens and HIV care[3, 4]. A 20-year-old HIV-positive adult on ART in the U.S. or Canada is expected to live into his early 70‘s, a life expectancy approaching that of the general population. However, large differences in life expectancy persist between certain sub-groups of patients according to sex, race, HIV transmission risk group, lifestyle and CD4 cell counts at ART initiation[3, 5, 6]. It is well-established that multimorbidity increases with age, moreover comorbidities, including cardiovascular diseases, diabetes, cancer, cognitive dysfunction, depression and osteopenia are more frequent in the HIV population[7–11]. Many age-related illnesses can be driven by HIV itself, and drug toxicities may play a role in specific organ systems and interact with medical conditions typically associated with advanced age[2, 12]. Due to the increased risk of age-related co-morbidities among HIV-positive adults, it is possible that life expectancy may plateau or decrease in the future. However, the concept of premature aging of HIV-infected persons appears to be controversial, particularly when considering the investigation of the effects of age on non–AIDS-defining malignancies[13].

Even if aging HIV cohorts included subjects aged 50 years and older, so far few of them have focused on geriatric population of HIV-infected patients aged 75 and over. It could be anticipated that the geriatric HIV population will have more co-morbidities and co-medications, much longer HIV infection, then more complex and/or less standard antiretroviral regimens, therefore a higher risk of not being virologically suppressed. The objective of this study was to assess to which extent the HIV geriatric population aged 75 and older differed from the HIV elderly population in terms of demographic and immuno-virological characteristics, ART history and current ART, and comorbidities.

## Material and methods

Dat’AIDS cohort is a French multicentric prospective cohort involving 19 HIV reference centres in France (Clinicaltrials.gov ref NCT02898987). These reference centres maintain prospective databases of all HIV infected patients seeking care in the centres and providing written consent. The data collection has been approved by the French national commission on informatics and liberty (CNIL). The database is implemented via an electronic medical[14]. Dat’AIDS cohort collects sociodemographic and clinical data, medical and ARV history, immunological and virological status at regular 3- to 6- month intervals during routine clinical assessment.

For the purpose of the study we selected all HIV patients aged 50 year-old and over at the last visit, with at least one visit since 2004 and still on follow-up at the censoring date (Dec 31^st^ 2015). Patients were classified as “elderly” if they were aged between 50 and 74 and “geriatric” if they were aged 75 or older at the latest visit. Medical history collected data on past or current diabetes (insulin-dependent or non insulin-dependent diabetes), hypertension (confirmed blood pressure above 140/90 mm Hg or hypertension treatment), dyslipidaemia (hypercholesterolemia and/or hypertriglyceridemia or treatment with lipid lowering agent), history of cardiovascular disease (ischemic cardiovascular disease or stroke), depression, and cancer. Renal insufficiency was defined by a confirmed estimated glomerular filtration rate below 60 ml/min/1.73 m^2^, as calculated using the Modification of Diet in Renal Disease (MDRD) formula.

Continuous variables were described by their medians and interquartile ranges (IQR) and compared between groups using a Mann-Whitney test. Categorical variables were described by proportions and compared by chi-square tests. R software version 3.0.2 was used for the analyses.

## Results

Among the 37,511 HIV infected adult patients included in the Dat’AIDS cohort with at least one visit since 2004 and still on follow-up at the 31 December 2015, 17 008 (45.3%) were older than 50 years (S1 Dataset). Among them 16,436 subjects were aged between 50 and 75 years and classified in the elderly group and 572 subjects were aged 75 years or older and classified in the geriatric group. The geriatric group represented 1.5% of the whole population. Socio-demographics and clinical characteristics of the elderly and geriatric groups are presented in Table 1. At the last visit, there was no difference in the proportion of HIV-1 infected subjects on ART, 97.3% and 97.2% in the geriatric and the elderly group, respectively, with a duration of known HIV infection slightly shorter in the geriatric group than in the elderly group (18.0 vs 19.0 years, p = 0.029) and a median duration of ART significantly longer in the geriatric group than in the elderly group, (16.0 vs 15.0 years, p = 0.003).

**Table 1:** Socio-demographic, clinical and immune-virological characteristics of the Elderly and Geriatric populations.

	Elderly 50–74		Geriatric ≥75		P. value
	n = 16 436		n = 572		
**Age,** median [IQR]	56	[52–61]	78	[76–81]	< 0.001
**Male,** n (%)	12,300	(74.8)	416	(72.7)	0.275
HBV and/or HCV Hepatitis coinfection*, n (%)	4,118	(25.1)	53	(9.3)	< 0.001
**HIV-2 infection,** n (%)	205	(1.2)	5	(0.9)	0.547
**HIV transmission,** n (%)					< 0.001
heterosexual	6,871	(44.7)	281	(55.4)	
MSM	6,057	(39.4)	194	(38.3)	
others	2,431	(15.8)	32	(6.3)	
**Country of birth,** n (%)					< 0.001
France	6,800	(64.5)	238	(68.0)	
African countries	1,811	(17.2)	32	(9.1)	
Others	2,308	(18.3)	80	(22.9)	
**Age at HIV diagnosis,** median [IQR]	39	[31–48]	61	[56–67]	<0.001
**<50 year-old,** n (%)	13,318	(81.3)	40	(7.0)	
**50–75 year-old,** n (%)	3,063	(18.7)	485	(85.1)	
**≥75 year-old,** n (%)	0	(0.0)	45	(7.9)	
**Time since HIV diagnosis, year,** med. [IQR]	19	[11–25]	18	[13–23]	0.029
**CDC stage C,** n (%)	4,713	(28.8)	192	(33.6)	0.015
**CD4/mm**^**3**^ **at diagnosis,** median [IQR]	300	[122–495]	246	[113–430]	0.039
**Nadir CD4 /mm3,** median [IQR]	183	[70–297]	159	[71–268]	<0.001
**BMI,** n (%)					< 0.001
underweight^1^	688	(4.3)	119	(21.6)	
normal weight^2^	9,596	(60.6)	390	(70.8)	
overweight^3^	4,142	(26.2)	30	(5.4)	
obesity^4^	1,402	(8.9)	12	(2.2)	
**25(OH)Vitamin D, ng/mL,** median [IQR]	31	[21–45]	30	[21–45]	0.716
**Serology CMV positive,** n (%)	10,443	(87.2)	373	(93.0)	< 0.001
**Current smoker,** n (%)	4,874	(38.7)	31	(7.5)	< 0.001
Alcohol abuse**, n (%)	2,980	(26.1)	83	(21.5)	0.049
**IVDU** current/past, n (%)	4,901	(38.6)	96	(23.0)	< 0.001
**At last visit**					
**CD4 /mm3,** median [IQR]	504	[324–720]	456	[304–634]	< 0.001
**CD4/CD8 ratio,** median [IQR]	0.6	[0.4–1.0]	0.6	[0.4–0.9]	0.137
**CD4≥500/mm3 and CD4/CD8≥1,** n (%)	2,452	(19.8)	74	(17.1)	0.178
** HIV RNA <50 c/mL, n(%)**	10,893	(89.8)	384	(89.9)	0.969
**HIV RNA <50 c/mL on ART**^5^**, n(%)**	10,739	(90.6)	378	(90.9)	0.932

BMI (Body Mass Index)

* Hepatitis B virus and/or Hepatitis C virus co-infection

^1^ <18 in Elderly, <21 in Geriatric

^2^ 18–25 in Elderly, 21–30 in Geriatric

^3^ 25–30 in Elderly, 30–35 in Geriatric

^4^ >30 in Elderly, >35 in Geriatric

^5^HIV-1 only

** More than 2 glasses of alcohol per day

At last visit, elderly patients were more frequently receiving a standard triple ART compared to of geriatric patients (74% and 68.2% respectively, p = 0.003), while proportion of patients receiving 4 or more ARVs and a dual therapy were 4.7% and 11.6% for the elderly group and2.7% and 16.5% for the geriatric grou, respectively (p = 0.042 and p <0.001). ART regimens at the last visit for elderly and geriatric HIV-1 infected patients are detailed in Table 2. Plasma HIV RNA was below 50 copies/mL in 90.6% and 90.9% of the subjects on ART in the elderly and geriatric group, respectively (p = 0.932).

**Table 2:** Current ART regimens in the elderly and geriatric groups (restricted to HIV-1 infected subjects).

Variable	Elderly (50–74)	Geriatric (≥75)	P. value*
On ART, n (%)	15,795 (96.1)	551 (96.3)	0.914
Duration on ART, year, median [IQR]	15 [8–19]	16 [10–19]	0.003
ART regimen			
Triple therapy, n (%)	11,692 (74.0)	376 (68.2)	0.003
2 NRTIs + 1 INSTI, n (%)	3,590 (22.7)	116 (21.1)	0.383
2 NRTIs + 1 PI, n (%)	258 (1.6)	16 (2.9)	0.034
2 NRTIs + 1 bPI, n (%)	2,845 (18.0)	77 (14.0)	0.018
2 NRTIs + 1 NNRTI, n (%)	4,894 (31.0)	156 (28.3)	0.198
3 NRTIs, n (%)	105 (0.7)	11 (2.0)	< .001
Monotherapy, n (%)	402 (2.5)	18 (3.3)	0.360
Monotherapy with bPI, n (%)	368 (2.3)	17 (3.1)	0.314
Other, n (%)	34 (0.2)	1 (0.2)	0.999
Dual therapy, n (%)	1,831 (11.6)	91 (16.5)	< .001
bPI + NNRTI, n (%)	220 (1.4)	6 (1.1)	0.678
bPI + INSTI, n (%)	376 (2.4)	14 (2.5)	0.920
bPI + LAM, n (%)	63 (0.4)	4 (0.7)	0.400
bPI + other, n (%)	182 (1.2)	6 (1.1)	0.999
INSTI + NNRTI, n (%)	613 (3.9)	31 (5.6)	0.050
2 NRTIs, n (%)	59 (0.4)	5 (0.9)	0.104
Other with no bPI, n (%)	318 (2.0)	25 (4.5)	< .001
4 drugs and more, n (%)	737 (4.7)	15 (2.7)	0.042

ART: antiretroviral therapy, PI: protease inhibitor, bPI: boosted protease inhibitor, NRTI: nucleoside/nucleotide reverse transcriptase inhibitor, NNRTI: non-nucleoside reverse transcriptase inhibitor, INSTI: integrase inhibitor, LAM: lamivudine

* Continuous variables were compared using a Mann-Whitney test. Categorical variables were compared by χ2 tests.

The elderly group had been less frequently exposed than the geriatric group to first-generation protease inhibitors and nucleoside reverse transcriptase inhibitors, 51.7% versus 59.0% (p<0.001) and 71.0% vs 79.9% (p<0.001), respectively. Exposure to non-nucleoside reverse transcriptase inhibitors and integrase inhibitors was similar between both groups (Table 3).

**Table 3:** ART exposure in the elderly and geriatric groups (restricted to HIV-1 infected subjects on ART).

Exposure to ARV and duration (months) in exposed patients	Elderly (50–74)			Geriatric (≥75)		P. value*
	n = 15,795			n = 551		
	N(%)	Median [IQR]		N(%)	Median [IQR]	
PI	13,205 (83.6)	94 [41–154]		461 (83.7)	94 [39–150]	0.999
First generation PI ^1^	8,170 (51.7)	49 [26–89]		325 (59)	45 [24–78]	< .001
LPV/RTV	5,721 (36.2)	38 [14–76]		202 (36.7)	33 [9–73]	0.868
DRV/RTV	5,690 (36)	41 [17–68]		164 (29.8)	44 [20–68]	0.003
ATV/RTV	5,499 (34.8)	55 [22–92]		183 (33.2]	67 [27–100]	0.465
NRTI	15,719 (99.5)	159 [93–218]		551 (100)	175 [110–226]	0.189
First generation NRTI^2^	11,213 (71)	97 [58–136]		440 (79.9)	107 [63–153]	< .001
TDF	12,849 (81.3)	73 [36–107]		387 (70.2)	69 [25–97]	< .001
ABC	9,076 (57.5)	58 [18–109]		370 (67.2)	66 [21–122]	< .001
NNRTI	11,897 (75.3)	62 [23–120]		423 (76.8)	74 [25–144]	0.468
Nevirapine	5,332 (33.8)	40 [9–110]		222 (40.3)	54 [11–139]	0.002
Efavirenz	6,810 (43.1)	41 [11–94]		226 (41)	43 [10–110]	0.350
Rilpivirine	2,570 (16.3)	20 [9–31]		75 (13.6)	11 [6–21]	0.108
Etravirine	2,521 (16)	39 [16–67]		72 (13.1)	51 [32–78]	0.077
INSTI^3^	6,999 (44.3)	28 [10–67]		253 (45.9)	36 [12–66]	0.483
Enfuvirtide	776 (4.9)	13 [5–28]		24 (4.4)	9 [6–15]	0.620

PI: protease inhibitor, LPV: lopinavir, DRV: darunavir, ATV: atazanavir, RTV: ritonavir, NRTI: nucleoside/nucleotide reverse transcriptase inhibitor, TDF: tenofovir DF, ABC: abacavir, ZDV: zidovudine, NNRTI: non-nucleoside reverse transcriptase inhibitor, INSTI: integrase inhibitor

^1^ RTV full dose, amprénavir, fosamprenavir, indinavir, nelfinavir, saquinavir, tipranavir

^2^zidovudine, didanosine, stavudine, zalcitabine

^3^ raltegravir, elvitegravir/cobicistat, dolutegravir

* Exposures were compared by χ^2^ tests

Co-morbidities were significantly more frequent in the geriatric group, except for dyslipidemia (Table 4). The most frequent comorbidities in the geriatric group were dyslipidemia (60.8%), hypertension (43.5%), chronic renal disease (29.4%) and cardiovascular disease (23.4%). In the geriatric group, 32.2% of the subjects had no more than one comorbidity, 49.5% had 2 or 3 comorbidities and 18.4% had 4 comorbidities or more, compared to 60.1%, 34.7% and 5.3%, respectively in the elderly group.

**Table 4:** Co-morbidities in the Elderly and Geriatric group.

Co-morbidity	Elderly (50–74)	Geriatric (≥75)	P.value*
	n = 16,436 N(%)	n = 572 N(%)	
Diabetes	1490 (9.1)	126 (22)	< .001
Hypertension	3452 (21)	249 (43.5)	< .001
Chronic renal disease	738 (4.5)	168 (29.4)	< .001
Dyslipidemia	9584 (58.3)	348 (60.8)	0.245
Cardiovascular disease	1775 (10.8)	134 (23.4)	< .001
Osteoporosis	1046 (6.4)	72 (12.6)	< .001
Depression	2933 (17.8)	80 (14)	0.020
Cancer	2026 (12.3)	131 (22.9)	< .001
Number of comorbidities			
0–1	9870 (60.1)	184 (32.2)	< .001
2–3	5701 (34.7)	283 (49.5)	
≥ 4	865 (5.3)	105 (18.4)	

* Co-morbidities were compared by χ^2^ tests

## Discussion

This study describes an aging HIV-infected population and because of the large population size we were able to compare an elderly population aged 50 to 74 years to a geriatric population aged 75 and over. To our knowledge our study is the first one to describe HIV and ART history, as well as comorbidities in a geriatric HIV population and shows that the high rate of virologic suppression is similar within the 2 groups despite more frequent comorbidities, a longer exposure to first generation protease inhibitors and thymidine analogs and more frequent non classical antiretroviral regimen in the geriatric group. Interestingly, the geriatric group had a significantly shorter duration of HIV infection than the elderly group, and had a significantly longer duration of antiretroviral therapy than the elderly population. However, these differences were only around 1 year, while difference in median age of the 2 groups was 22 years, suggesting that the geriatric group either acquired HIV infection at an already advanced age (93% of the geriatric subjects were diagnosed after 50 years old and among them 7.9% after 75 years old), or was diagnosed late after many years of latent chronic HIV infection (median age at HIV diagnosis of 61 years). Indeed, the geriatric group had a significantly lower nadir CD4 cell count and a higher prevalence of CDC stage C and a median age at HIV diagnosis of 61 years. Of note, and in contrast with the elderly group, geriatric patients had acquired HIV mainly through heterosexual contact, which could contribute to a lower perception of the risk of HIV infection and to increased delay for HIV testing. Studies on missed opportunities for HIV diagnosis have identified old age and being heterosexual has significant risk factors for being diagnosed late [15, 16]. Although the geriatric population had been more frequently exposed to first-generation ARVs, including protease inhibitors and thymidine analogue nucleoside reverse transcriptase inhibitors, they were less likely to receive complex regimens consisting of 4 ARVs or more and more likely to receive a dual therapy. This suggests that with newer ARVs, the need for complex antiretroviral regimens consisting of multiple drugs because of cumulative or cross-resistance is decreasing. On the other hand, because of a higher prevalence of co-morbidities, a higher number of co-medications, or an age-related chronic renal impairment, adaptation of treatment to avoid cumulative toxicities or drug-drug-interactions is probably more frequently needed in geriatric patients, which could explain the more frequent use of dual therapy in this population. Use of nucleosidic- and PI- sparing regimens to avoid the cumulative toxicity of antiretroviral therapy represents a major issue, particularly in aging subjects highly ART experienced, confronted with lipodystrophy, renal, cardio-vascular and other co-morbidities. Dual therapy has been shown to be a possible switch option in virologically suppressed patients, as long as certain conditions are fulfilled[17]. Some of these dual therapies have been evaluated among them combination of an integrase inhibitor plus a non nucleosidic reverse transcriptase inhibitor, lamivudine or maraviroc with a benefit on lipid profile and bone mineral density[18–20]. The combination of one integrase inhibitor plus one non nucleosidic reverse transcriptase inhibitor was the dual therapy most frequently prescribed in the geriatric group. Recent data of the ANRS163-ETRAL study showed the robust and potent activity with an excellent safety profile of the dual therapy raltegravir plus etravirine in subjects over 45 years virologically suppressed and with a long history of antiretroviral therapy and frequent lipodystrophy[21]. The dual therapy in maintenance of dolutegravir plus either rilpivirine or lamivudine have also shown reassuring results both on efficacy and renal tolerability, with the advantage of a simple once-daily regimen[22] [18]. With the development of these new promising PI-sparing and/or NRTI-sparing switch dual therapy, future studies need to address their specific benefit in HIV geriatric patients. Interestingly, not only older age but also a high number of comorbidities was driving the choice for a mono/dual therapy. These data suggest that physicians are more and more concerned about prevention of comorbidities and long-term toxicities.

In the general population, co-morbidities are increasing with age and are not linear but rather significantly accelerate at older age. Our result is consistent with the study of Guaraldi at al. where 14% of patients aged 60 year-old and over had 4 comorbidities or more[23]. Some studies have shown a higher and earlier frequency of these comorbidities in the HIV population compared with the general population[5, 24]. Some of these comorbidities may be induced or worsened by ART. In our study geriatric patients had been more often exposed to first generation antiretrovirals including oldest PI favouring metabolic syndrome, and increasing cardiovascular risk and thymidine analogs responsible for lipoatrophy and mitochondrial toxicity. In the Dutch AgeHIV cohort with a median age of 52.4 years, prevalence of hypertension is high (46.2%) and the authors show that changes in body composition, involving both abdominal obesity and stavudine-induced peripheral lipoatrophy, might contribute to the higher prevalence of hypertension in HIV-1-infected patients [25]. Neurocognitive dysfunction, obstructive pulmonary disease, and osteoporosis, have also been linked to low nadir CD4+ T-cell counts[26].

In the centers from which the data were drawn, the proportion of individuals aged 50 years and over on ART and with HIV RNA < 50 c/mL on treatment is very similar to the global population (96% and 89.9% vs 94.3% and 87.4%, respectively (n = 35890, personal data). On ART, despite a high rate of virological success above 90% and a long duration on ART above 15 years, quality of immune reconstitution (considered as optimal if CD4 count is above 500/mm3 and CD4:CD8 ratio above 1) was poor in this aging population both in the elderly and the geriatric groups with only 19.8% and 17.1% of the subjects reaching both more than 500 CD4 cell/mm3 and a CD4:CD8 ratio above 1, respectively, as it was pointed out in some studies [27]. Many similarities on immunological alterations have been observed between middle-aged HIV-infected individuals and non-HIV geriatric subjects[28]. It is of importance to point out that very few elderly patients have been included in studies assessing HIV aging and the relative impact of HIV infection and other parameters on inflammatory and immune disorders has not yet been fully studied in this population[29]. Hentzien *et al*. studied the impact of age related morbidities on on five-year overall mortality in an aging population aged 60 and over. They showed that age-related comorbidities—particularly cardiovascular diseases and chronic renal disease—were the main prognostic factors for mortality, at the same weight as CD4 cell count[30]. In this context of highly experienced elderly patients, our study shows that the choice of ARV was driven by presence of co-morbidities as well as prevention of long-term toxicities without impairing virologic suppression. Polymedication is a major issue in the geriatric and elderly populations, with the risk of increased adverse drug events, drug-drug interactions, inappropriate medications and poor adherence [31]. In a recent study, Greene *et al*.show that HIV subjects older than 60 were at high risk of polypharmacy and medications related problems and that the overall burden of medications has shifted from antiretrovirals to comorbidities-associated medications and points out the need for a geriatric-sensitive care of the ageing HIV population [32]. The multidisciplinary approach that is recommended for ART management including physicians, virologists and pharmacists to optimize HIV infection management should integrate geriatricians for the patients that enter elderly age [33, 34].

Our study has some limitations; some comorbidities may have been underestimated because of a non declaration in the patient’s medical chart. However results of a recent French study evaluating comorbidities in HIV patients over 60 years find similar prevalence of the major comorbidities (cardiovascular diseases 30%, hypertension 27% and diabetes 15%) [35]. Furthermore individuals who died before Dec 31, 2015 were not included in the study and this does not allow to evaluate the frequency and reasons of death in this aging population.

In summary, our study points out that a geriatric HIV population is emerging and highlights the burden and challenge of this geriatric HIV population who despite more co-morbidities and more advanced HIV infection achieve similar high rate of virologic suppression than the elderly population. A systematic multidisciplinary approach, involving general practitioners, infectiologists, geriatricians, pharmacists should be developed to face the incoming challenge of HIV-infected population advancing to geriatric age.

## Supporting information

S1 Dataset Anonymous dataset of the study. (XLS)
